# P-1173. Analysis of MIC and Disk Diffusion Testing Variables for Gepotidacin and Levofloxacin against Gram-Positive and Gram-Negative Isolates

**DOI:** 10.1093/ofid/ofaf695.1366

**Published:** 2026-01-11

**Authors:** Nicole E Scangarella-Oman, Laura M Koeth, Jeanna M DiFranco-Fisher, Josh West

**Affiliations:** GlaxoSmithKline plc., Collegeville, PA; Laboratory Specialists, Inc., Westlake, OH; Laboratory Specialists, Inc., Westlake, OH; GSK, Collegeville, Pennsylvania

## Abstract

**Background:**

Gepotidacin is a novel, bactericidal, first-in-class triazaacenaphthylene antibacterial that selectively inhibits bacterial replication through a distinct binding site, unique mechanism of action and, for most target pathogens, well-balanced inhibition of two Type II topoisomerase enzymes (DNA gyrase and topoisomerase IV). Gepotidacin was recently approved by the FDA for the treatment of uncomplicated urinary tract infections (uUTI). This study evaluated the effects of several antimicrobial susceptibility testing (AST) variables on standardized (i.e., CLSI, EUCAST) testing methods for key uUTI uropathogens.

**Figure:**
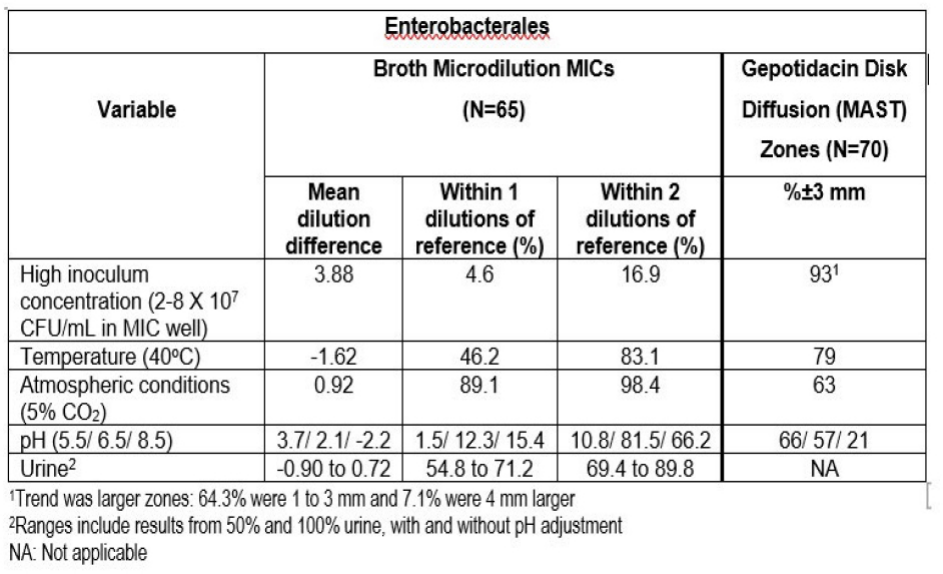
Variables shown to significantly impact gepotidacin BMD MICs and/or DD zone for Enterobacterales

**Methods:**

The in vitro activity of gepotidacin and levofloxacin was evaluated using 4 CLSI AST methods: broth microdilution (BMD), broth macrodilution (MD), agar dilution (AD) and disk diffusion (DD). 85 clinical isolates were included for MIC testing and 90 isolates for DD, including *E. faecalis* (EF), *S. saprophyticus* (SS), and several Enterobacterales species known to be implicated in uUTI. Variables (BMD and DD only) including but not limited to inoculum concentration, temperature, atmospheric conditions, pH, and urine were tested.

**Results:**

Gepotidacin mean reference BMD MICs varied no more than 1 dilution over multiple days, for all species tested. MICs were comparable between the 3 MIC testing methods with MICs for 84 of 85 isolates within ± 1 dilution. Reference gepotidacin mean disk results varied by approximately 1-2 mm over multiple testing days. Mueller Hinton agar data was similar for DD with the exception of SS and Remel in which the majority of results were > 3mm larger. Variables shown to significantly impact gepotidacin BMD MICs and/or DD zone for Enterobacterales are shown in the table below.

Results were similar for SS and EF with the exception of EF and SS DD zones being more impacted by inoculum variation and no impact of temperature on EF and SS MICs and DD zones.

**Conclusion:**

When performing susceptibility testing with gepotidacin it is important to control the following variables, which were shown in this study to have the most impact on results: high inoculum concentration, high temperature (for Enterobacterales only), pH, incubation in 5% CO2 (for DD only) and testing of BMD MICs in urine.

**Disclosures:**

Nicole E. Scangarella-Oman, MS, GSK: Employee|GSK: Stocks/Bonds (Public Company) Josh West, BS, GSK: Employee|GSK: Stocks/Bonds (Public Company)

